# *In vivo* Expansion of Naïve CD4^+^CD25^high^ FOXP3^+^ Regulatory T Cells in Patients with Colorectal Carcinoma after IL-2 Administration

**DOI:** 10.1371/journal.pone.0030422

**Published:** 2012-01-20

**Authors:** Marc Beyer, Beatrix Schumak, Martin R. Weihrauch, Bettina Andres, Thomas Giese, Elmar Endl, Percy A. Knolle, Sabine Classen, Andreas Limmer, Joachim L. Schultze

**Affiliations:** 1 Life and Medical Sciences Institute, Genomics and Immunoregulation, University of Bonn, Bonn, Germany; 2 Institute of Medical Microbiology, Immunology and Parasitology (IMMIP), University of Bonn, Bonn, Germany; 3 Clinic I for Internal Medicine, University of Cologne, Cologne, Germany; 4 Institute of Immunology, University of Heidelberg, Heidelberg, Germany; 5 Institute for Molecular Medicine and Experimental Immunology, University of Bonn, Bonn, Germany; Wayne State University, United States of America

## Abstract

Regulatory T cells (T_reg_ cells) are increased in context of malignancies and their expansion can be correlated with higher disease burden and decreased survival. Initially, interleukin 2 (IL-2) has been used as T-cell growth factor in clinical vaccination trials. In murine models, however, a role of IL-2 in development, differentiation, homeostasis, and function of T_reg_ cells was established. In IL-2 treated cancer patients a further T_reg_-cell expansion was described, yet, the mechanism of expansion is still elusive. Here we report that functional T_reg_ cells of a naïve phenotype - as determined by CCR7 and CD45RA expression - are significantly expanded in colorectal cancer patients. Treatment of 15 UICC stage IV colorectal cancer patients with IL-2 in a phase I/II peptide vaccination trial further enlarges the already increased naïve T_reg_-cell pool. Higher frequencies of T-cell receptor excision circles in naïve T_reg_ cells indicate IL-2 dependent thymic generation of naïve T_reg_ cells as a mechanism leading to increased frequencies of T_reg_ cells post IL-2 treatment in cancer patients. This finding could be confirmed in naïve murine T_reg_ cells after IL-2 administration. These results point to a more complex regulation of T_reg_ cells in context of IL-2 administration. Future strategies therefore might aim at combining IL-2 therapy with novel strategies to circumvent expansion and differentiation of naïve T_reg_ cells.

## Introduction

Human regulatory T cells (T_reg_ cells) have been characterized as CD4^+^CD25^high^ T cells with inhibitory function [1]. They are crucial for the preservation of T-cell homeostasis and self-tolerance and regulate the immune responses to alloantigens, pathogens and tumors [2]. Both in humans and animal models activation of T_reg_ cells results in exertion of their full suppressive function [3], [4]. Natural T_reg_ cells are generated in the thymus as a distinct lineage of anergic CD4^+^ T cells bearing self-reactive T-cell receptors, although cells with similar characteristics can also be generated in the periphery under appropriate conditions [5]. Typically, T_reg_ cells express cytotoxic T-lymphocyte–associated-antigen 4 (CTLA-4) and glucocorticoid-induced tumor-necrosis-factor receptor-related protein (GITR), although both molecules can also be expressed by activated T cells [6], [7]. The transcription factor forkhead box P3 (FOXP3) has been demonstrated to be expressed exclusively on T_reg_ cells in the mouse [2], while data concerning its expression in humans are not as clear-cut [8], [9]. In numerous murine tumor models increased frequencies of CD4^+^CD25^high^ T_reg_ cells seem to be a hallmark of tumor progression and metastasis [10], [11]. Moreover, efficient anti-tumor immune responses are induced by deletion of these cells resulting in complete tumor regression [12], [13]. In humans, we and others have demonstrated that CD4^+^CD25^high^FOXP3^+^ T_reg_ cells are also expanded in patients with solid tumors and hematologic malignancies and contribute to the overall immunosuppression in these patients [14], [15]. Numerous animal models over the last years could demonstrate that increased numbers of T_reg_ cells are beneficial for tumor growth while depletion of T_reg_ cells can lead to tumor regression [16]. In humans, administration of an IL-2 immunotoxin to tumor patients results in decreased numbers of T_reg_ cells and higher responses against simultaneously administered tumor peptides [17], [18]. Several studies over the last years have addressed the question of T_reg_-cell frequencies in colorectal cancer patients [19], [20], [21]. These reports could demonstrate increased number of FOXP3^+^ T_reg_ cells in the peripheral blood, tumor-draining lymph nodes and in close tumor proximity [19], [20], [21]. Still, the question why T_reg_ cells are expanded in human tumors remains elusive.

CD45RO expression has been primarily linked to T_reg_ cells, which led to the assumption that T_reg_ cells belong to the memory T-cell compartment [4], [22]. Recently, a T_reg_-cell population with a naïve phenotype (CCR7^+^CD45RA^+^) was identified in healthy individuals [23], [24]. These naïve T_reg_ cells proliferated vigorously in response to auto-antigens suggesting that particularly this subpopulation was specific for self rather than foreign antigens [23]. Persistence of naïve T_reg_ cells has been described throughout adult life [25], [26], although it must be pointed out that this pool of naïve T_reg_ cells is relatively small in peripheral blood of healthy individuals [27]. In patients with multiple myeloma (MM), however, we could demonstrate an expansion of naïve T_reg_ cells [28]. We could validate this finding for a number of hematologic malignancies and patients with solid tumors and even show strong suppressive function for the naïve T_reg_-cell population [29]. Comparison of levels of T-cell receptor excision circles (TREC) in T_reg_ cells of healthy individuals addressed the diversity and developmental stage of thymic emigrants as well as peripheral blood T_reg_ cells [30], [31]. As expected, TREC numbers were significantly higher in thymic emigrants compared to peripheral blood derived T_reg_ cells, which supports thymic development of human CD4^+^CD25^high^ T_reg_ cells [31].

On the one hand, IL-2 is both important for the development and expansion of effector T cells and also critical in the context of immune tolerance [32]. On the other hand, experiments performed in murine models established that IL-2 and its downstream effector molecules are essential for the generation, maintenance, and function of T_reg_ cells [33], [34], [35], [36]. Several studies have addressed the role of IL-2 administration on the frequency and function of human T_reg_ cells in cancer patients and demonstrated that treatment with recombinant IL-2 induces an expansion of T_reg_ cells in peripheral blood, thereby interfering with efficient anti-tumor immune responses [37], [38], [39], [40], [41], [42], [43]. These studies pointed to a peripheral expansion of T_reg_ cells post IL-2 therapy [41]. It was further suggested that altered migratory behavior due to increased expression of CCR4 and CXCR4 on T_reg_ cells might be associated with peripheral expansion and increased migration to the tumor site [41]. In two recent studies however, Correale *et al.* demonstrated that the combination of chemotherapy with GM-CSF and IL-2 administration leads to reduced T_reg_-cell numbers [44], [45]. These findings suggested a more complex regulation of T_reg_-cell frequency, distribution and function than previously thought. Moreover, it remains unclear whether different mechanisms might be responsible for frequency changes of T_reg_ cells in these different patient populations treated with IL-2.

In the current study, we investigated the impact of IL-2 administration on the frequency and function of CD4^+^CD25^high^FOXP3^+^ T_reg_ cells. We provide clear evidence for an increased expansion of naïve T_reg_ cells particularly post IL-2 therapy as a major mechanism of overall T_reg_-cell expansion in these cancer patients. Our data clearly suggest an increase of newly generated T_reg_ cells in cancer patients. These novel findings are of particular interest for strategies targeting T_reg_ cells in cancer patients.

## Results

### Increased frequencies of CD4^+^CD25^high^FOXP3^+^ T_reg_ cells in peripheral blood of patients with metastatic colorectal cancer

**Figure 1:**
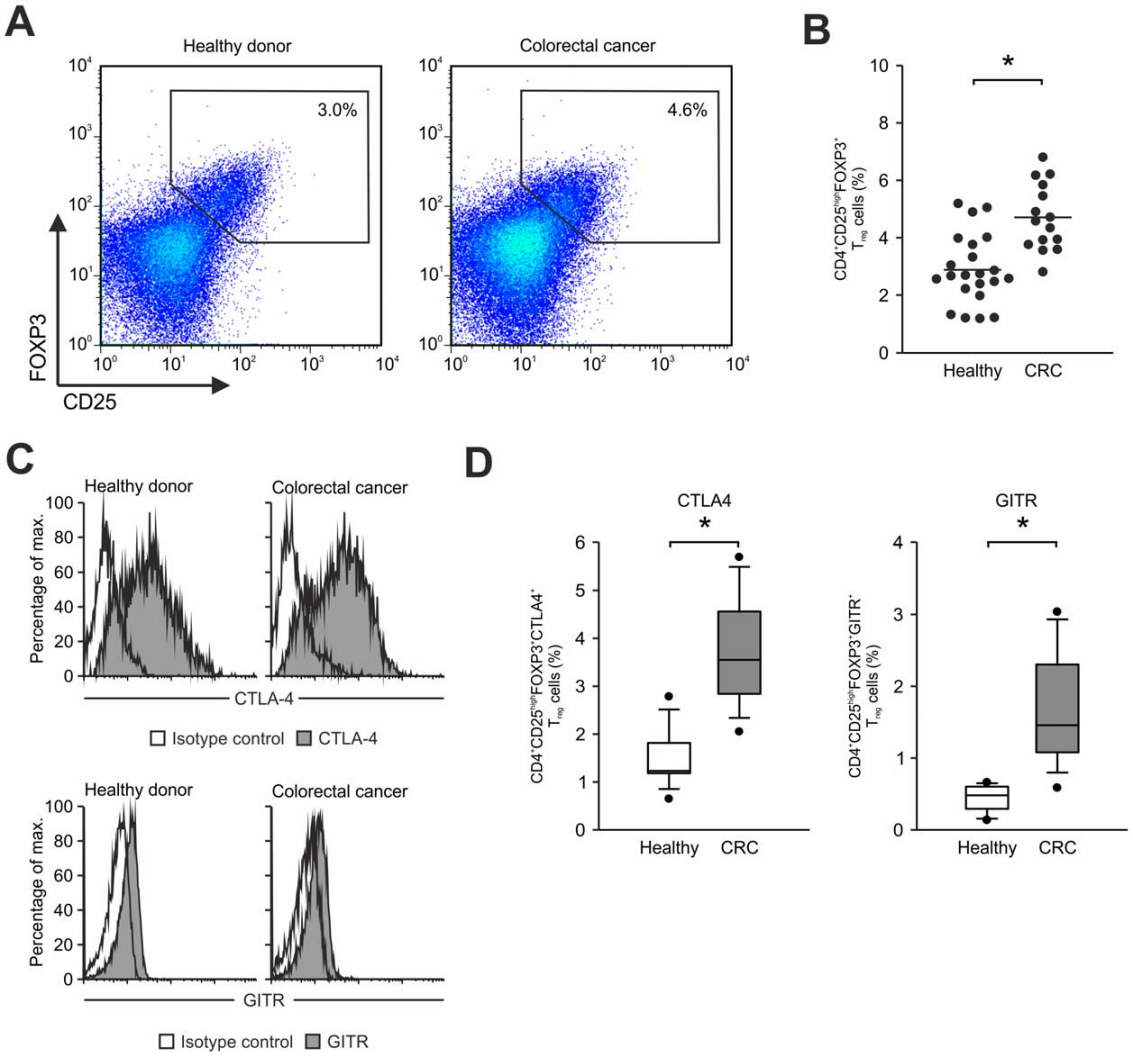
Frequency of CD4^+^CD25^high^FOXP3^+^ T_reg_ cells. (**A**) Flow cytometric analysis of CD25 and FOXP3 on peripheral blood derived CD4^+^ T cells from a representative healthy individual (left panel) and a representative colorectal cancer patient before treatment (right panel). Numbers represent percentage of events within the gate. (**B**) Frequency of CD4^+^CD25^high^FOXP3^+^ T_reg_ cells in 22 healthy donors and 15 colorectal cancer patients (CRC) before treatment. Each dot represents a single individual assessed in the respective group; mean expression (line) of all samples in each group is also shown (*, p<0.05, Student's *t* test). (**C**) CTLA4 (top) and GITR expression (bottom) in CD4^+^CD25^high^FOXP3^+^ T_reg_ cells of healthy donors (left, grey fill) and colorectal cancer patients (right, grey fill). Isotype control (black line). (**D**) Frequency of CTLA4 (left) and GITR (right) expressing CD4^+^CD25^high^FOXP3^+^ T_reg_ cells in healthy donors (white) and colorectal cancer patients (grey, CRC) before treatment. Shown here are median, 25^th^ and 75^th^ percentile (box), 10^th^ and 90^th^ percentile (whiskers) and outliers (dots), (*, p<0.05, Student's *t* test).

Within a clinical phase I/II combined chemoimmunotherapy trial of patients with metastatic colorectal cancer we assessed frequencies of FOXP3-expressing CD4^+^CD25^high^ T_reg_ cells in peripheral blood before initiation of therapy in comparison to healthy controls (Fig. 1A). The frequency of T_reg_ cells in healthy donors (n = 22, 2.9%±1.2%) was comparable to previously published results (Fig. 1B) [46], [47]. In contrast, individuals with colorectal cancer assessed before initiation of treatment (n = 15, 4.7%±1.2%, p<0.001) showed significantly increased frequencies of T_reg_ cells compared to healthy individuals (Fig. 1A and 1B). We also assessed previously described surface receptors associated with T_reg_ cells including CTLA4 and GITR on CD4^+^CD25^high^FOXP3^+^ T cells in colorectal cancer patients as well as healthy individuals. As depicted in Figure 1C, we observed comparable expression of both molecules on T_reg_ cells from healthy donors and colorectal cancer patients while percentages of CD4^+^CD25^high^FOXP3^+^ T_reg_ cells expressing either molecule were significantly increased in colorectal cancer patients (CTLA4: 3.7%±1.2% vs. 1.5%±0.6%, p<0.001; GITR: 1.7%±0.8% vs. 0.5%±0.2%, p<0.001) (Fig. 1D).

### Expansion of CD4^+^CD25^high^FOXP3^+^ T_reg_ cells after chemoimmunotherapy including low-dose IL-2 administration

**Figure 2:**
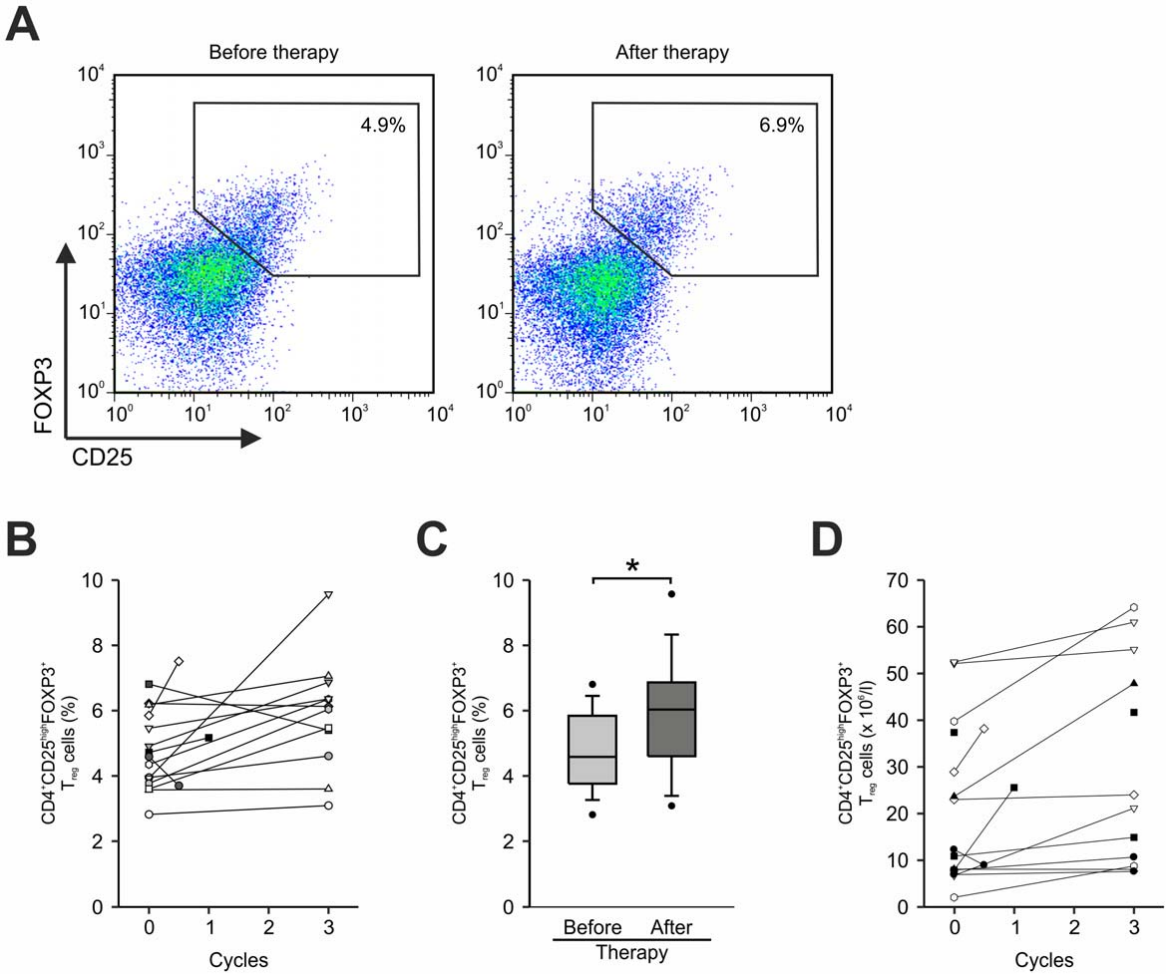
Analysis of frequencies of CD4^+^CD25^high^FOXP3^+^ T_reg_ cells after chemoimmunotherapy. (**A**) Flow cytometric analysis of CD25 and FOXP3 on peripheral blood derived CD4^+^ T cells from a representative colorectal cancer patient before (left panel) and after chemoimmunotherapy (right panel). Numbers represent percentage of events within the gate. (**B**) Serial analysis of frequencies of CD4^+^CD25^high^FOXP3^+^ T_reg_ cells in colorectal cancer patients before and after chemoimmunotherapy. (**C**) Frequency of CD4^+^CD25^high^FOXP3^+^ T_reg_ cells in 15 colorectal cancer patients before (light grey box) and after (dark grey box) chemoimmunotherapy. Shown here are median, 25^th^ and 75^th^ percentile (box), 10^th^ and 90^th^ percentile (whiskers) and outliers (dots), (*, p<0.05, Student's *t* test). (**D**) Serial analysis of total numbers of CD4^+^CD25^high^FOXP3^+^ T_reg_ cells in colorectal cancer patients before and after chemoimmunotherapy.

Next, we investigated changes in frequency of T_reg_ cells in respect to IL-2 administration (Fig. 2). From the 15 patients included in the trial, 12 successfully completed the whole protocol, while 3 patients were rapidly progressing under therapy and deceased shortly thereafter. Blood samples from the 12 patients (P1–12) who completed the whole treatment were taken after completion of IL-2 treatment following vaccination. For the 3 rapidly progressing patients (P13–15) who were taken off study, T_reg_-cell frequencies were assessed at the last time point were material was available. As depicted for patient P11 in Figure 2A we observed an expansion of CD4^+^CD25^high^FOXP3^+^ T_reg_ cells in the majority of patients. When assessing all patients, 11 colorectal cancer patients had higher frequencies post therapy while 2 (P3, P15) had lower frequencies at the second time point of analysis and two other patients (P4, P5) showed similar frequencies at both time points (Fig. 2B). No feature (laboratory test, treatment or clinical parameter) we have assessed so far showed an association with changes in T_reg_-cell frequency in these patients (data not shown). In addition, we did not observe any cytopenia greater grade 2 during therapy. Overall, in the majority of patients the frequency of T_reg_ cells after combined chemoimmunotherapy was increased compared to the initial frequencies before treatment (5.8%±1.7% vs. 4.7%±1.2%, p<0.05) (Fig. 2C) as well as in comparison to healthy donors (5.8%±1.7% vs. 2.9%±1.2%, p<0.001). When assessing the absolute numbers of T_reg_ cells we observed the same trend; total numbers of T_reg_ cells were increased after chemoimmunotherapy (after: 29.2×10^6^/l±20.5×10^6^/l vs. before: 21.3×10^6^/l±17.1×10^6^/l, p<0.005) and only one of the rapidly progressing patients showed reduced numbers of T_reg_ cells (Fig. 2D).

Among the 15 colorectal cancer patients, 4 patients had progressive disease at the time of analysis while 11 showed a response or stable disease. Comparing these two patient cohorts revealed no significant difference in the proportion of T_reg_ cells, whereas both patient cohorts showed higher frequencies of T_reg_ cells compared to healthy controls (data not shown). Next, we assessed if T_reg_-cell frequencies were indicative of longer freedom from treatment failure or overall survival or if these 2 parameters were linked to the expansion of T_reg_ cells, yet no statistically significant correlation was detected (data not shown). Our data indicate that an expansion of CD4^+^CD25^high^FOXP3^+^ T_reg_ cells occurred in the majority of colorectal cancer patients after IL-2 administration as part of combined chemoimmunotherapy.

### Inhibitory function of CD4^+^CD25^high^ T_reg_ cells from colorectal cancer patients

**Figure 3:**
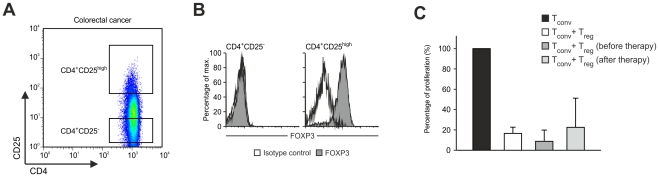
Functional analysis of CD4^+^CD25^high^FOXP^+^ T_reg_ cells. (**A**) CD4^+^ cells were separated by flow cytometric cell sorting into conventional CD4^+^CD25^−^ and regulatory CD4^+^CD25^high^ T cells as defined by their expression of CD25. (**B**) Re-analysis of FOXP3 expression in CD4^+^CD25^−^ T_conv_ (left, grey fill) and CD4^+^CD25^high^ T_reg_ cells (right, grey fill) post cell sorting. Isotype control (black line). (**C**) Reduction of proliferation of CD4^+^CD25^−^ T_conv_ cells stimulated with beads coated with CD3 and CD28 mAbs (black bar) by highly purified CD4^+^CD25^high^FOXP3^+^ T_reg_ cells from healthy donors (white bar) or colorectal cancer patients before (dark grey bar) and after therapy (light grey bar).

In 4 colorectal cancer patients sufficient numbers of highly purified CD4^+^CD25^high^ T_reg_ cells (Fig. 3A and B) were isolated from peripheral blood by flow cytometric cell sorting before and after vaccination to analyze their inhibitory function in comparison to T_reg_ cells from healthy controls (n = 4). Proliferation of allogeneic conventional CD4^+^CD25^−^ T cells stimulated by beads coated with CD3 and CD28 mAbs was used as the read out to assess inhibitory function of CD4^+^CD25^high^ T_reg_ cells [28]. Proliferation of allogeneic conventional CD4^+^CD25^−^ T cells was significantly inhibited when highly purified CD4^+^CD25^high^ T cells from healthy donors were added at a 1∶1 ratio (white bar, Fig. 3C, p<0.001). On a cell-to-cell basis highly purified CD4^+^CD25^high^ T_reg_ cells from colorectal cancer patients (before initiation of therapy) showed an equally strong inhibitory function on conventional CD4^+^CD25^−^ T-cell proliferation (dark grey bar, Fig. 3C, p<0.001). Titration experiments demonstrated that the inhibitory function of T_reg_ cells from healthy individuals and colorectal cancer patients was comparable also at lower T_reg_∶T_conv_ ratios (data not shown). Moreover, after IL-2 treatment of colorectal cancer patients (after treatment), T_reg_ cells had equal suppressive function on conventional CD4^+^CD25^−^ T-cell proliferation when compared to T_reg_ cells isolated before start of therapy (light grey bar, Fig. 3C, p<0.001). Taken together, these data suggest, that T_reg_ cells from colorectal cancer patients have normal suppressive function.

### Expansion of fully functional naïve T_reg_ cells in colorectal cancer patients after chemoimmunotherapy

**Figure 4:**
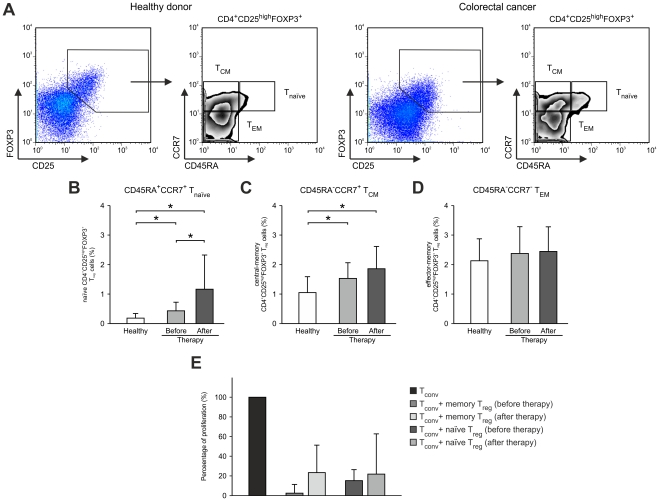
Increase of naïve CD4^+^CD25^high^FOXP3^+^ T_reg_ cells in colorectal cancer patients after chemoimmunotherapy. (**A**) Strategy of flow cytometric analysis of CCR7 and CD45RA expression on the surface of CD4^+^CD25^high^FOXP3^+^ T_reg_ cells as exemplified for a representative healthy donor (left) and a representative colorectal cancer patient (right). Frequencies of (**B**) CCR7^+^CD45RA^+^ naïve T_reg_ cells (T_naive_), (**C**) CCR7^+^CD45RA^−^ central memory T_reg_ cells (T_CM_), and (**D**) CCR7^−^CD45RA^−^ effector memory T_reg_ cells (T_EM_) were assessed in peripheral blood of colorectal cancer patients (n = 15) before (light grey bars) and after therapy (dark grey bars) as well as healthy individuals (white bars, n = 22). Significant differences (p<0.05, Student's *t* test) between healthy donors and colorectal cancer patients before and after chemoimmunotherapy are marked by an asterisk (*). Error bars represent SD. (**E**) Assessment of regulatory function of naïve and memory CD4^+^CD25^high^ T_reg_ cells sorted according to their CD45RA expression from colorectal cancer patients. Reduction of proliferation of CD4^+^CD25^−^ T_conv_ cells stimulated with beads coated with CD3 and CD28 mAbs by highly purified naïve and memory CD4^+^CD25^high^ T_reg_ cells from colorectal cancer patients before and after therapy.

It has been previously reported by us and others that differential expression of the cell surface receptors CD45RA and CCR7 can be used to differentiate between naïve, central and effector memory T_reg_ cells (Fig. 4A) [23], [28], [48]. This approach can be used to address the question, if naïve, central or effector memory T_reg_ cells contribute to the overall expansion of T_reg_ cells in patients with solid tumors and how treatment, particularly administration of IL-2 influences the frequency and function of naïve versus memory CD4^+^CD25^high^FOXP3^+^ T_reg_ cells. In healthy individuals, naïve CCR7^+^CD45RA^+^ T_reg_ cells were hardly detectable (Fig. 4B). T_reg_ cells were almost exclusively of a memory phenotype (Fig. 4C and D) with a higher frequency of T_EM_ cells (Fig. 4D). In contrast, in colorectal cancer patients we observed a significantly higher number of T_reg_ cells with a CCR7^+^CD45RA^+^ naïve phenotype (Fig. 4B). This was further accompanied by an increase of T_reg_ cells with a central memory phenotype in colorectal cancer patients prior to therapy (Fig. 4C) while effector memory T_reg_ cell levels were comparable in colorectal cancer patients (prior to therapy) and healthy donors (Fig. 4D). However, most surprising, after IL-2 treatment, expansion of T_reg_ cells almost exclusively occurred within the naïve T_reg_-cell population (Fig. 4B) while frequencies of central and effector memory T_reg_ cells remained unchanged (Fig. 4C and D). To further characterize the increased subset of naïve CD4^+^CD25^high^FOXP3^+^ T_reg_ cells, we assessed the expression of intracellular CTLA4 or GITR expression. Comparable to the data obtained for the total T_reg_-cell population both molecules were expressed at similar levels on a per cell basis on naïve T_reg_-cells from healthy individuals and cancer patients irrespective of IL-2 treatment (data not shown) while percentages of CD4^+^CD25^high^FOXP3^+^ T_reg_ cells expressing either molecule were significantly increased in colorectal cancer patients (CTLA4: 0.31%±0.23% vs. 0.05%±0.01%, p<0.05; GITR: 0.10%±0.07% vs. 0.02%±0.01%, p<0.05) with a further increase after IL-2 administration (CTLA4: 0.78%±0.56% vs. 0.31%±0.23%, p<0.001; GITR: 0.24%±0.19% vs. 0.10%±0.07%, p<0.05) (Fig. S1).

Next, we assessed the suppressive function of naïve versus memory T_reg_-cell populations before and after therapy (Fig. 4E). We observed a clear inhibition of conventional CD4^+^CD25^−^ T-cell proliferation induced by stimulation with CD3/CD28-coated beads by adding sorted T_reg_ cells to the culture irrespective of the subtype (naïve vs. memory) used or the status of therapy (no therapy vs. chemoimmunotherapy) demonstrating that the different subtypes of T_reg_ cells have full suppressive activity independent of IL-2 therapy. Taken together, these data demonstrate a significant expansion of naïve T_reg_ cells post IL-2 treatment. Furthermore, these cells are similarly effective in suppressing conventional T-cell activation when analyzed on a cell-to-cell basis, suggesting an overall higher suppressive effect of these cells in colorectal cancer patients.

### Thymic expansion of naïve CD4^+^CD25^high^FOXP3^+^ T_reg_ cells in patients with colorectal cancer after IL-2 administration

**Figure 5:**
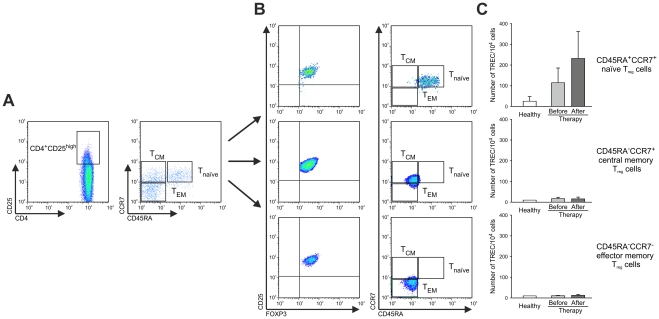
Replicative history of CD4^+^CD25^high^FOXP3^+^ T_reg_ cell populations defined by the expression of CD45RA and CCR7. CD4^+^CD25^high^ T_reg_ cells were isolated by flow cytometric cell sorting according to their expression of CD25 as well as CD45RA and CCR7 in three T_reg_-cell subsets, namely T_naïve_ (CD45RA^+^CCR7^+^), T_CM_ (CD45RA^−^CCR7^+^), and T_EM_ cells (CD45RA^−^CCR7^−^). (**A**) Strategy of flow cytometric analysis of CD4 and CD25 expression on the surface of CD4^+^ T cells as exemplified for a colorectal cancer patient. (**B**) Re-analysis of FOXP3 and CD25 expression (left) as well as CCR7 and CD45RA expression (right) in CD4^+^CD25^high^ T_reg_ cells. (**C**) Naïve, central and effector memory CD4^+^CD25^high^ T_reg_ cells from healthy donors and colorectal cancer patients before and after chemoimmunotherapy were assessed for TREC (T-cell receptor excision circle) content. Genomic DNA of sorted subsets was isolated, and the number of TREC was determined by quantitative real-time PCR. Data are shown as the mean values obtained for 2 independent healthy donors and 2 colorectal cancer patients. Error bars represent SD.

As T_reg_ cells with a naïve phenotype were increased in patients with colorectal cancer, particularly post IL-2 treatment, we were interested to assess whether the increase of T_reg_ cells resulted from peripheral expansion or possibly thymic generation of CD4^+^CD25^high^FOXP3^+^ T_reg_ cells. We previously reported that the level of TREC can be used as a marker to estimate the developmental vicinity of T_reg_ cells to the thymus and their division history [28] and therefore applied TREC analysis to address this issue in relation to IL-2 treatment. PB derived CD4^+^ T cells from two colorectal cancer patients and two age-matched healthy individuals were sorted according to their CD25, CCR7 and CD45RA expression into the appropriate CD4^+^CD25^high^ T_reg_-cell subsets, namely T_naïve_, T_CM_, and T_EM_ (Fig. 5A and B). TREC values for the highly purified T_reg_-cell subsets were assayed by real-time PCR. As described before, CD4^+^CD25^high^ T_reg_ cells showed relatively low TREC contents in the naïve T-cell population and TREC levels were below detection threshold in the T_CM_ and T_EM_ subsets (Fig. 5C) [28]. These observations are in line with the concept of antigen-driven peripheral expansion of T_reg_ cells in healthy individuals rather than recent thymic emigration. In contrast, the TREC content on the single cell level in naïve CD4^+^CD25^high^ T_reg_ cells in colorectal cancer patients was more than two-fold higher in average compared to healthy individuals before initiation of chemoimmunotherapy and even more increased after administration of IL-2 (>4–fold in average, Fig. 5C). These results strongly suggest that the expansion of T_reg_ cells in colorectal cancer patients was nurtured by the generation of new T_reg_ cells in the thymus and the further increase of T_reg_-cell frequencies post IL-2 administration was strongly associated with an increase of thymic emigrants of naïve T_reg_ cells.

### Administration of IL-2 leads to an expansion of a population of murine “naïve” CD4^+^ CD25^high^FOXP3^+^ T_reg_ cells

**Figure 6:**
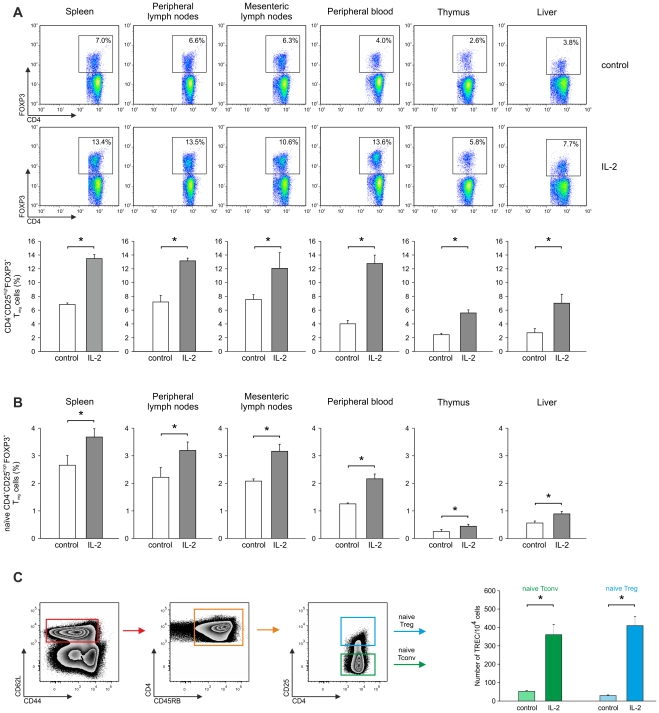
IL-2 administration leads to an expansion of “naïve” CD4^+^CD25^high^FOXP3^+^ T_reg_ cells in C57BL/6 mice. (**A**) Flow cytometric analysis of CD4 and FOXP3 expression in CD4^+^ T cells from untreated as well as IL-2-treated animals in spleen, peripheral and mesenteric lymph nodes, peripheral blood, thymus, and liver. Significant differences (p<0.05, Student's *t* test) between untreated and IL-2 treated animals are marked by an asterisk (*). (**B**) Analysis of “naïve” CD45RB^high^ CD4^+^CD25^high^FOXP3^+^ T_reg_ cells in spleen, peripheral and mesenteric lymph nodes, peripheral blood, thymus, and liver. Significant differences (p<0.05, Student's *t* test) between untreated and IL-2 treated animals are marked by an asterisk (*). Similar results were obtained in two independent experiments. (**C**) CD45RB^+^CD44^low^CD62L^+^ naïve CD4^+^CD25^−^ T_conv_ and CD4^+^CD25^high^ T_reg_ cells were isolated by flow cytometric cell sorting and assessed for TREC content. Genomic DNA of sorted subsets was isolated, and the number of TREC was determined by quantitative real-time PCR (n = 3, p<0.05, Student's *t* test). Error bars represent SD. Similar results were obtained in three independent experiments.

To demonstrate that IL-2 administration indeed leads to an increase of naïve T_reg_ cells, we administered IL-2 or PBS i.p. in C57BL/6 mice for 10 days and assessed the frequency of CD4^+^CD25^high^FOXP3^+^ T_reg_ cells in these animals after the treatment period. In comparison to PBS-treated animals a significant expansion of CD4^+^CD25^high^FOXP3^+^ T_reg_ cells occurred after IL-2 administration in spleen, peripheral as well as mesenteric lymph nodes, peripheral blood, thymus, and liver (Fig. 6A). Of particular interest was the increase of T_reg_ cells in the thymus, which could be indicative of an increased thymic output. As a first approximation for vicinity of T cells to thymic output expression of CD45RB on CD4^+^CD25^high^FOXP3^+^ T_reg_ cells was assessed since up to now no true marker for murine naïve T_reg_ cells has been established. We observed a significant increase of CD45RB^high^ CD4^+^CD25^high^FOXP3^+^ T_reg_ cells in spleen, peripheral as well as mesenteric lymph nodes, peripheral blood, thymus, and liver of IL-2 treated mice (Fig. 6B) clearly indicating that an increase of naïve T_reg_ cells contributed to the overall expansion of T_reg_ cells after IL-2 administration and that the overall T_reg_-cell expansion was related to elevated frequencies of naïve T_reg_ cells in the thymus.

To further delineate if IL-2 treatment induces generation of naïve T_reg_ cells in the thymus we sorted CD45RB^+^CD44^low^CD62L^+^ naïve T_conv_ and T_reg_ cells from IL-2 as well as PBS treated animals (Fig. 6C) and assessed TREC levels in the naïve T_conv_ and T_reg_-cell population. We observed significantly higher levels of TREC in T_conv_ and T_reg_-cell populations after IL-2 administration (Fig. 6C) suggesting that IL-2 treatment indeed results in a higher thymic output of naïve T_reg_ cells as observed in IL-2 treated human colorectal cancer patients.

## Discussion

In the current study we addressed the question whether IL-2 treatment leads to an expansion of fully functional T_reg_ cells in colorectal cancer patients and whether an increase of T_reg_ cells in colorectal cancer patients is due to peripheral or thymic expansion. Using phenotypic, functional and molecular approaches we demonstrate an increase of CD4^+^CD25^high^FOXP3^+^ T_reg_ cells in colorectal cancer patients already before initiation of an IL-2 containing chemoimmunotherapy regimen. The increase of T_reg_ cells in these patients was further augmented after completion of treatment, clearly demonstrating that IL-2 treatment is associated with an increase in T_reg_-cell numbers an alarming effect which could potentially influence and dampen the immune response in an immunotherapy protocol. T_reg_ cells expressed phenotypic markers associated with T_reg_ cells (e.g. CTLA-4 and GITR) and suppressed the proliferation of CD4^+^CD25^−^ T_conv_ cells. Furthermore, we can clearly show that T_reg_ cells in colorectal cancer patients have normal suppressive function.

In addition, we demonstrate that the expansion of T_reg_ cells in colorectal cancer patients was particularly prominent within the naïve CCR7^+^CD45RA^+^ CD4^+^CD25^high^FOXP3^+^ T_reg_ cell population and to a lesser extent in central memory T_reg_ cells, while there was no difference in T_reg_ cells with an effector memory phenotype. Most surprising, expansion of T_reg_ cells post IL-2 treatment was almost exclusively due to an increase of naïve T_reg_ cells. Expansion of naïve T_reg_ cells was further supported by the increase of TREC numbers that were significantly higher in naïve T_reg_ cells from untreated colorectal cancer patients when compared to healthy controls and further increased post IL-2 treatment. To demonstrate that the observed results in humans are dependent on the IL-2 administration, we treated mice with IL-2 and could detect increased levels of T_reg_ cells in these animals. In line with our results in humans, we observed thymic generation of naïve T_reg_ cells after IL-2 treatment as shown by high TREC levels in the naïve T_reg_-cell population. These findings strongly suggest that T_reg_-cell expansion in colorectal cancer patients is due to higher thymic output which is further increased by IL-2 treatment.

Over the last years several murine studies showed the importance of IL-2 for T_reg_ cells. IL-2 is a critical growth factor for murine T_reg_ cells, it is responsible for maintenance and regulation of T_reg_ cells in the periphery [33], [34], [35]. IL-2 seems to be involved in the generation of T_reg_ cells during antigen-specific immune responses [49] and has been suggested to be involved in the suppressive function of T_reg_ cells [50], [51]. In human T_reg_-cell biology, IL-2 is supposed to induce a peripheral expansion of CD4^+^CD25^high^FOXP3^+^ T_reg_ cells as suggested by Wei *et al.* [41] while it has no effect on FOXP3 expression in conventional T cells. This has been further confirmed in a second study which reported that IL-2 induced STAT-dependent mechanisms are responsible for the selective expression of FOXP3 in T_reg_ cells and *in vivo* expansion following IL-2 administration indicating a unique programming of CD4^+^CD25^high^ T_reg_ cells within the IL-2 signaling pathway [52].

Most recently increased frequencies of CD4^+^CD25^high^FOXP3^+^ T_reg_ cells were reported for patients with renal cell carcinoma, malignant melanoma or ovarian cancer patients after IL-2 monotherapy [37], [39], [40], [41] and administration of IL-2 during immune reconstitution after chemotherapy in pediatric sarcomas led to a preferential expansion of T_reg_ cells after cytoreductive chemptherapy [38]. In contrast, patients with metastatic colorectal cancer treated with a combined chemoimmunotherapy containing gemcitabine and FOLFOX-4 (oxaliplatin, fluorouracil, and folinic acid) polychemotherapy followed by the subcutaneous administration of GM-CSF and low-dose IL-2 showed clinical objective responses in the majority of patients associated with a significant reduction in CD4^+^CD25^high^FOXP3^+^ T_reg_ cells as reported by Correale *et al.* [44], [45].

Our data however support an alternative outcome of combined chemoimmunotherapy as low-dose IL-2 in combination with a peptide-vaccination resulted in increased frequencies of CD4^+^CD25^high^FOXP3^+^ T_reg_ cells, particularly naïve T_reg_ cells.

These rather opposite results might be explained by the differences in the vaccination and chemotherapy protocols, e.g. high-dose vs. low-dose 5-FU, irinotecan vs. oxaliplatin, forgoing of gemcitabine, dosage and schedule of GM-CSF administration, or addition of CAP-1-peptide, in the time course of administration, in the patient cohort under study (metastatic colorectal cancer vs. unselected colorectal cancer), number of patients analyzed, and additional, yet unknown, confounding factors. Further conflicting might be technical issues as it has been previously stated by Baecher-Allan *et al.* that the assessment of human T_reg_ cells is still difficult and the use of different assays sometimes makes it difficult to compare different studies [47]. Indeed, a recent study in patients with renal cell carcinoma or malignant melanoma suggested an enormously high proportion of naïve T_reg_ cells [39]. However, the number of naïve T_reg_ cells in the healthy control group was also reported to exceed 50% of all T_reg_ cells, a frequency that could never be confirmed by us and others [24], [27].

Irrespective of these experimental differences in earlier studies, we clearly unraveled the expansion of naïve T_reg_ cells to be one of the mechanisms leading to an overall expansion of fully functional T_reg_ cells in colorectal cancer patients which was further augmented by IL-2 therapy. Over the last years differentiation of T_reg_ cells into naïve, central and effector memory T_reg_ cells according to their expression of CCR7 and CD45RA has been established for healthy individuals [23], [24], [25], [31], [53]. We have incorporated this strategy for the analysis of T_reg_ cells in cancer patients and demonstrated an expansion of naïve T_reg_ cells in human multiple myeloma patients and B-CLL patients [28]. Remarkably, in B cell malignancies, increase of T_reg_ cells was associated with peripheral expansion of naïve T_reg_ cells while in patients with colorectal cancer the expansion seems to be thymus dependent as determined by analysis of TREC as an approximation of adjacency of T_reg_ cells to the thymus. Moreover, administration of IL-2 further augmented this increase. Two recent publications underlined the importance of IL-2 for the development of T_reg_ cells in the thymus [54], [55], while other results support a more restricted role for IL-2 of regulating T_reg_ cells in the periphery without alteration of the thymic output [34]. The latter proposition has been further strengthened by data obtained by Wei *et al.* who could demonstrate expansion of memory T_reg_ cells *in vitro* by IL-2 treatment [41]. To address whether effects induced by IL-2 were limited to peripheral expansion of mainly memory T_reg_ cells, we treated C57BL/6 mice with IL-2 and assessed the frequencies of T_reg_ cells with a special focus on naïve T_reg_ cells in the periphery and in the thymus. We observed an overall expansion of T_reg_ cells in these animals and detected a particular enrichment of naïve T_reg_ cells in the thymus and peripheral lymph nodes. By assessment of TREC in the naïve T_reg_-cell population we could demonstrate thymic generation of naïve T_reg_ cells as a result of IL-2 adminstration, clearly suggesting that increased thymic output is an important mechanism leading to increased frequencies of naïve T_reg_ cells post IL-2 treatment and these cells subsequently can differentiate into peripheral T_reg_ cells with a memory phenotype. Whether peripheral expansion might also occur under these conditions might be further studied by administration of IL-2 to thymectomized animals.

The assessment of the source of expanded T_reg_ cells in cancer patients is of particular importance as the mechanism of expansion, an augmented production of T_reg_ cells in the thymus, expansion of T_reg_ cells in the periphery, preferential migration of T_reg_ cells to the tumor site, increased conversion of conventional T cells into T_reg_ cells as well as diminished apoptosis and cell death of peripheral T_reg_ cells, might influence the strategy to therapeutically target T_reg_ cells to increase anti-tumor immunity. E.g. peripherally expanded T_reg_ cells might be deleted by short-term use of cytotoxic agents such as denileukin diftitox while continuing thymic expansion would require either long-term treatment or deletion of the cause of thymic expansion of these inhibitory cells to induce a long-lasting reduction of T_reg_ cells. Other mechanisms such as conversion of differentiated conventional T cells into T_reg_ cells might also contribute to the overall expansion of T_reg_ cells in cancer patients as well the preferential egress of activated Treg cell from the tumor microenvironment into the peripheral blood and will have to be addressed when targeting T_reg_ cells [5].

Taken together, we demonstrate an *in vivo* expansion of fully functional CD4^+^CD25^high^FOXP3^+^ T_reg_ cells in colorectal cancer patients due to an increase in naïve T_reg_ cells with an increased TREC content. Moreover, naïve T_reg_ cells with a further increase of TREC are expanded post IL-2 treatment clearly pointing to an increased thymic output of naïve T_reg_ cells after IL-2 therapy, a mechanism also observed in mice *in vivo* post IL-2 treatment. This expansion of T_reg_ cells post administration of IL-2 can potentially hinder an immune response towards co-administered anti-tumor reagents and should therefore be avoided when planning new cancer immunotherapy protocols. The existence of different mechanisms of expansion of T_reg_ cells (thymic vs. peripheral expansion and conversion) highlights the complexity of regulation of these cells and cautions the use of simple strategies targeting these highly regulated cells in future cancer immunotherapy approaches.

## Materials and Methods

### Patients and clinical parameters

15 HLA-A2^+^ patients with primary metastatic colorectal cancer were enrolled in this Phase I/II combined chemoimmunotherapy with a HLA-A2 peptide derived from the carcinoembryonic antigen CEA (CAP-1) and irinotecan, 5-fluorouracil, and leucovorin after approval by the institutional review committee at the University of Cologne and the German Drug Administration [56]. All patients signed informed consent. Mean age was 56.2±13.1 years; 8 were male and 7 were female; 12 had colon while 3 had rectal cancer. Mean age for the corresponding healthy controls was 49.5±9.3 years with no significant differences in gender and age. Inclusion criteria required an age of between 18 and 75 years, positive HLA-A2 status, elevated serum CEA (>5 µg/l) and/or CEA-positive tumor, untreated metastatic disease, chemotherapy-free interval after adjuvant treatment of at least 6 months, Karnofsky index >70%, life expectancy of at least 3 months, sufficient bone marrow and liver function, HIV and hepatitis B and C negativity, absence of central nervous system metastases, no immunosuppressant medication, and negative pregnancy test. All patients received low-dose IL-2 (1×10^6^ IU) post vaccination. For vaccination, several approaches were compared in this study, namely a vaccine containing only the CAP-1-peptide, or the CAP-1-peptide together with 50 µg GM-CSF (Novartis) or the CAP-1-peptide together with an oligonucleotide adjuvant (dSLIM, Mologen); as a fourth option, patients obtained autologous CAP-1-pulsed dendritic cells as a cellular vaccine. For the analysis of T_reg_-cell frequency and function post IL-2 treatment, no statistically significant influence of the different types of vaccinations could be established (data not shown). Two cycles of vaccination and IL-2 treatment were given 2 and 1 week before the first cycle of chemotherapy consisting of 80 mg/m^2^ irinotecan, 2,000 mg/m^2^ high-dose 5-FU, and 500 mg/m^2^ leucovorin (six weekly administrations). Alternating, two cycles of IL-2 and vaccination were combined with one cycle of chemotherapy and repeated three times. After the third cycle, patients were treated with IL-2 and CAP-1-peptide vaccination on a weekly schedule until progressive disease. At baseline and after three cycles of chemoimmunotherapy, patients underwent a leukapheresis to obtain PBMC for diagnostic and therapeutic purposes. PBMC were isolated using Ficoll/Hypaque (Amersham, Uppsala, Sweden) density centrifugation. Staging was performed according to the UICC classification for colorectal cancer. All patients were UICC stage IV. The treatment schedule is provided as Figure S2. Characteristics of the patients studied are summarized in Table 1.

**Table 1:** patient characteristics.

Patient ID	Sex	Age(y)	Primary tumor	Sites of metastases	Primary vaccine	Boost	time point of 2nd analysis	Clinical response	FFTF (mo)	Survival (mo)
P01	M	66	Colon	Liver	CAP-1+GM-CSF+IL-2	CAP-1+IL-2	after 3 cycles	PR	15	34
P02	F	44	Colon	Liver	CAP-1+dSLIM+IL-2	CAP-1+dSLIM+IL-2	after 3 cycles	SD	5	21
P03	F	70	Colon	Liver	CAP-1+dSLIM+IL-2	CAP-1+dSLIM+IL-2	after 3 cycles	SD	12	32
P04	F	32	Colon	Liver, spleen, ovaries, pelvis, peritoneum	DC-CAP-1+IL-2	CAP-1+dSLIM+IL-2	after 3 cycles	CR	12	26
P05	F	60	Colon	Liver	CAP-1+IL-2	CAP-1+IL-2	after 3 cycles	SD	11	15
P06	M	57	Rectum	Lung	CAP-1+dSLIM+IL-2	CAP-1+dSLIM+IL-2	after 3 cycles	SD	7	17
P07	M	44	Rectum	Liver	CAP-1+dSLIM+IL-2	CAP-1+dSLIM+IL-2	after 3 cycles	CR	12	28
P08	M	64	Colon	Liver, lung	DC-CAP-1+IL-2	CAP-1+dSLIM+IL-2	after 3 cycles	SD	10	28
P09	F	43	Rectum	Liver, bone, pararectal, paraaortal lymphnodes	CAP-1+GM-CSF+IL-2	CAP-1+GM-CSF+IL-2	after 3 cycles	PD	6	13
P10	M	61	Colon	Liver	CAP-1+dSLIM+IL-2	CAP-1+dSLIM+IL-2	after 3 cycles	CR	9	11
P11	M	62	Colon	Peritoneum	CAP-1+IL-2	CAP-1+IL-2	after 3 cycles	CR	13	21
P12	M	55	Colon	Liver (primary resection)	CAP-1+dSLIM+IL-2	CAP-1+dSLIM+IL-2	after 3 cycles	CR	24	24
P13	F	38	Colon	Liver	DC-CAP-1+IL-2	CAP-1+dSLIM+IL-2	after 1 cycle	PD	3	10
P14	F	72	Colon	Liver, spleen, lung, mediastinum	CAP-1+IL-2	CAP-1+IL-2	after 0.5 cycle	PD	2	2
P15	M	75	Colon	Liver	CAP-1+IL-2	CAP-1+IL-2	after 0.5 cycle	PD	1	6

### Antibodies and FACS analysis

Phenotype of T cells was defined by flow cytometry using the following antibodies: CD4-FITC, CD45RA-PE-Cy-5, CD4-APC, CD4-APC-Cy-7 (all from Becton Dickinson PharMingen), CD25-PE-Cy7 (BDBiosciences), CCR7-FITC (R&D Systems) as well as the corresponding isotype control antibodies (BDPharMingen). Intracellular staining was performed with the following antibodies: FOXP3-PE or APC (eBioscience), GITR-FITC (R&D Systems), CTLA4-PE or with the appropriate isotype controls (BDPharMingen) [28], [46]. Cells were stained according to the manufacturer's recommendations.

Samples were acquired on a FACSCanto and analyzed with FlowJo software (TreeStar Inc.). Frequencies of CD4^+^CD25^high^FOXP3^+^ T cells are shown as percent values of CD4^+^ T cells.

### Isolation of CD4^+^CD25^high^ and CD4^+^CD25^−^ T cells

For functional analysis, CD4^+^CD25^high^ T cells were purified from PBMC. Briefly, CD4 MACS Beads (Miltenyi Biotec) were used for isolation of CD4^+^ T cells [28], [46]. After staining with CD25-PE, and CD4-APC (BDPharMingen) according to the manufacturer's recommendations, CD4^+^CD25^high^ T cells were purified using a FACSDiVa Cell Sorter (BDBiosciences) and used for functional characterization. The CD4^+^CD25^−^ T cells isolated from healthy individuals were used as effectors to assess T_reg_ cell function independently of potential defects of conventional CD4^+^ T cells from colorectal cancer patients [28], [46]. The cells were re-analyzed for FOXP3 expression after sorting and routinely showed >95% purity.

### Assessment of inhibitory function

To assess the suppressive activity of T_reg_ cells, 5,6-Carboxyfluorescin-Diacetat-Succinimidyl-Ester (CFSE, Sigma-Aldrich) stained CD4^+^CD25^−^ T cells (5×10^4^/well) were stimulated with magnetic beads (Dynal Biotech) coated with 5% anti-CD3 (OKT3), 14% anti-CD28 (9.3) and 81% anti-MHC class I (W6/32) at a ratio of 3∶1 (cells∶beads) in X-VIVO 15 supplemented with 10% fetal calf serum, 100 U/ml penicillin/streptomycin and 2 mM glutamine (all from Invitrogen). PKH-26 (Sigma-Aldrich)-labelled allogeneic CD4^+^CD25^high^ T cells or naive or memory CD4^+^CD25^high^ T cells activated for 20 hours with 10 U/ml IL-2 (Proleukin®, Chiron) and 0.5 µg/ml anti-CD3 mAb in X-VIVO 15 (BioWhittakker) were added at a 1∶1 ratio to the culture and proliferation of CD4^+^CD25^−^ T cells was determined by assessing CFSE dilution after four days of culture [28].

### Isolation of CD4^+^CD25^high^ T-cell subpopulations for assessment of T-cell receptor excision circles and functional characterization

Briefly, CD4 MACS Beads were used for isolation of CD4^+^ T cells [46]. After staining with CCR7-FITC, CD25-PE, CD45RA-PE-Cy-5, and CD4-APC, CD4^+^CD25^high^ T cells and the respective T cell subsets, CCR7^+^CD45RA^+^ T_naïve_, CCR7^+^CD45RA^−^ T_CM_, and CCR7^−^CD45RA^−^ T_EM_ cells were purified using a FACSDiVa Cell Sorter and used for either functional characterization or assessment of TREC levels.

DNA was isolated from purified CD4^+^CD25^−^, CD4^+^CD25^low^ and CD4^+^CD25^high^ T_naïve_, T_CM_, and T_EM_ cells respectively using a DNA Isolation Kit (Roche Diagnostics) following the manufacturer's instructions.

Relative TREC levels were determined using real-time PCR with a LightCycler (Roche Diagnostics) based on specific primers and general fluorescence detection with SYBR Green. All PCR were performed using LightCycler-FastStart DNA Master SYBR Green I kit (Roche Diagnostics). All samples were studied in duplicate reactions using the human TREC primer kit (Search-LC). The number of TREC molecules in the sample was calculated as number of copies per 10^4^ cells (detection limit ≥10 molecules).

### Analysis of IL-2 effects on murine T_reg_ cells

Female C57BL/6 mice of 7 weeks were obtained from Elevage Janvier (France) and maintained in our animal facility. *In vivo* experiments were approved by the Animal Care Commission of Nord-Rhein-Westfalia, Germany (TVZ 9.93.2.10.31.07.089). After either treatment with human IL-2 (1×10^5^ IU/mouse/day, Proleukin) i.p. or PBS as control for 10 days [57], mice were sacrificed and their spleens, thymi, liver, and LN removed. Single-cell suspensions were prepared and stained for flow cytometric analysis. Antibodies used for staining were CD4-PE-Cy7, CD8α-Pacific Blue, CD45RB-APC-Cy7, CD25-Alexa 647, and FOXP3-Alexa 488 as well as appropriate isotype controls (all from BDBiosciences or BioLegend). Samples were acquired on a FACSCantoII and analyzed with FlowJo software. Frequencies of CD4^+^CD25^high^FOXP3^+^ T_reg_ cells are shown as percent values of CD4^+^ T cells.

### Isolation of CD4^+^CD45RB^+^CD44^low^CD62L^+^ naïve murine T-cells for assessment of T-cell receptor excision circles

Briefly, CD4 MACS Beads were used for isolation of murine CD4^+^ T cells from the spleen after treatment with IL-2 as described above [46]. After staining with CD44-FITC, CD62L-PE, CD25-Alexa 647, CD45RB-APC-Cy-7, CD8α-PE-Cy-7, and CD4-Alexa 405, naïve CD45RB^+^CD44^low^CD62L^+^ conventional CD4^+^CD25^−^ T cells as well as naïve CD4^+^CD25^+^ T_reg_ cells were purified using a FACSDiVa Cell Sorter and used for assessment of TREC levels.

Murine δRec-ψJα TRECs were determined using real-time quantitative polymerase chain reaction (PCR) as described before [58]. Briefly, cells were centrifuged and the pellet frozen at −80°C until analysis. DNA was isolated from purified naïve CD4^+^CD25^−^ T_conv_ and naïve CD4^+^CD25^high^ T_reg_ cells using a DNA Isolation Kit (Roche Diagnostics) following the manufacturer's instructions. Relative TREC levels were determined using real-time PCR containing mδRec primer (5′-GGGCACACAGCAGCTGTG), ψJα primer (5′-GCAGGTTTTTGTAAAGGTGCTCA), and mδRec-ψJα fluorescent probe (5′-FAM-CACAAGCACCTGCACCCTGTGCA-TAMRA-3′). Lysates were separately subjected to amplification of the single-copy CD8β chain gene using a CD8β forward primer (5′-CAGGACCCCAAGGACAAGTACT-3′), CD8β reverse primer (5′-CACTTTCACCATACAAAACTCCTTTG-3′), and CD8β probe (5′-FAMTGAGTTCCTGGCCTCCTGGAGTTCTTC-TAMRA-3′). Reactions contained 0.5 µM of each primer, 0.3 µM fluorescent probe, and Platinum Quantitative PCR Supermix-UDG (Invitrogen) Amplifications were performed in triplicate on an LightCycler 480 II (Roche Diagnostics). Amplification conditions were 50°C for 2 minutes, 95°C for 5 minutes, then 40 cycles of 95°C for 15 seconds, and 60°C for 1 minute. Standards for murine TRECs (mδREC-ψJα) and CD8β were provided by Dr. Y.-W. Chu (Center for Cancer Research, National Institutes of Health). Standard curves were generated as described before [58]. TREC frequency (TREC molecules per 10 000 cells) was determined by normalizing the number of TRECs amplified in the real-time PCR reaction to the number of amplified CD8β molecules.

### Statistical analysis

Comparison between paired or unpaired groups was performed using the appropriate Student's *t*-test. A p-value<0.05 was defined as statistically significant. All statistical analyses were performed using the SPSS statistical software package (SPSS 19, SPSS Inc.). Figures were created using SigmaPlot 12.0 (Systat Software Inc).

## Supporting Information

Figure S1 **CTLA4 and GITR expression in naïve T_reg_ cells.** Frequency of CTLA4 (left) and GITR (right) expressing naïve CCR7^+^CD45RA^+^ CD4^+^CD25^high^FOXP3^+^ T_reg_ cells in healthy donors (white) and colorectal cancer patients before (light grey) and after IL-2 administration (dark grey) before treatment. Shown here are median, 25^th^ and 75^th^ percentile (box), 10^th^ and 90^th^ percentile (whiskers) and outliers (dots), (*, p<0.05, Student's *t* test).
(TIF)

Figure S2 **Therapy schedule.** Patients were first randomized to receive CAP-1 and IL-2 with different adjuvants (dSLIM, GM-CSF, or none). Subsequently, they were randomized to receive their first vaccination with or without pulsed autologous dendritic cells. Vaccinations (V) and chemotherapy (Chemo) were given in an alternating schedule, starting with two vaccinations.
(TIF)
