# Supplementary figures and images for: In vivo Expansion of Naïve CD4+CD25high FOXP3+ Regulatory T Cells in Patients with Colorectal Carcinoma after IL-2 Administration
Source: PLoS One. 2012 Jan 20;7(1):e30422. doi: 10.1371/journal.pone.0030422 (PMC3262821; doi:10.1371/journal.pone.0030422)

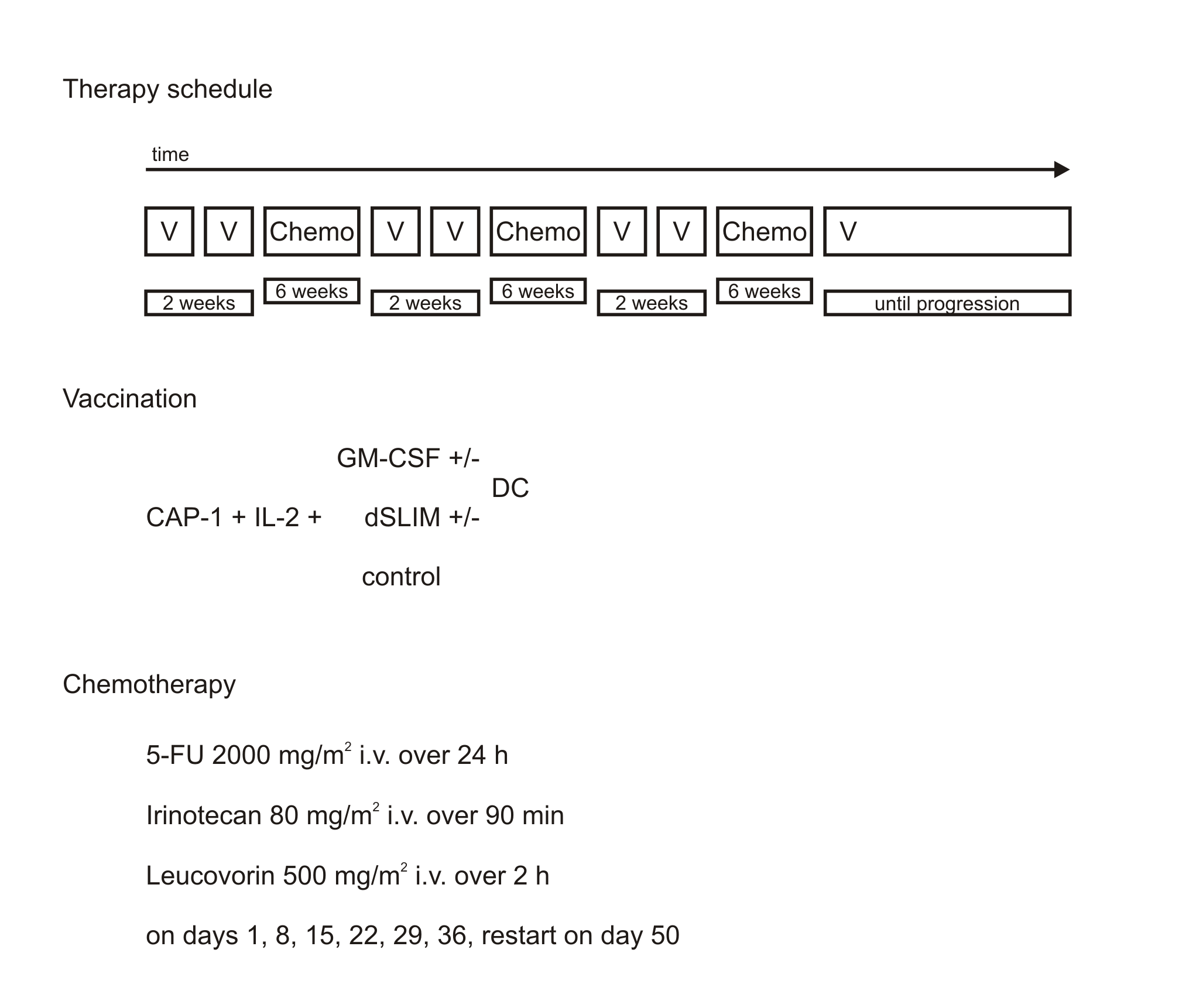

Supplement: Figure S2 — Therapy schedule. Patients were first randomized to receive CAP-1 and IL-2 with different adjuvants (dSLIM, GM-CSF, or none). Subsequently, they were randomized to receive their first vaccination with or without pulsed autologous dendritic cells. Vaccinations (V) and chemotherapy (Chemo) were given in an alternating schedule, starting with two vaccinations. (TIF) [file pone.0030422.s002.tif]
